# Supplementary material for: A qualitative study of knowledge, attitudes and perceptions towards malaria prevention among people living in rural upper river valleys of Nepal
Source: PLoS One. 2022 Mar 18;17(3):e0265561. doi: 10.1371/journal.pone.0265561 (PMC8932613; doi:10.1371/journal.pone.0265561)
Supplement: S1 File — (PDF) [file pone.0265561.s001.pdf]

Interview guide for qualitative study on malaria

**Interview Code**	
**Interviewee Code**	

**Name of Interviewer**	
**Date of Interview**	
**Time Started**	
**Time Finished**	
**Location**	
**Age of Interviewee**	
**Sex of Interviewee**	

Interview Domains of Enquiry

(Introduction)

- **Could you please tell us a bit about yourself and your family?**

(Decision making and health care)

- **Could you tell me about how you take care of yourself or your family member when you are sick?**
- **Could you share a time when you made a decision about health care or treatment while you were sick?**
- **Could you describe the decision-making process for health in the family?**
- **Would you describe where you seek care and with whom when you fall sick?**
- **Would you describe a visit to a health personnel/ doctor/ traditional healer for a check-up for us?**
- **Could you tell me about the services and treatments you received at the health facility/ private medical/ traditional healer and your experience?**

(Disease specific)

- **Could you please tell us what you know about malaria?**
- **Could you share you or your family's experience of malaria?**
- **Do you think malaria can be severe?**

(Risk and Prevention)

- **What are some of the ways malaria can be prevented?**

- Now since we have talked about the various ways of prevention in general, now I want to explore what YOU do, are they the same steps you and your family would take? If, yes can you tell me why?
- Can you share with me what activities has the government or local body taken on malaria prevention in your village?
    - Prompts
        - IRS
        - LLIN
- Can you share your experience regarding use of LLINs/ bed nets?

मलेरियाको गुणात्मक अध्ययनको लागि अन्तर्वार्ता गाईड

अन्तर्वार्ता कोड	
अन्तर्वार्ताकर्ताको कोड	

अन्तर्वार्ताकर्ताको नाम	
अन्तर्वार्ताको मिति	
समय (सुरु)	
समय (सकियो)	
स्थान	
अन्तर्वार्ता दिनेको उमेर	
अन्तर्वार्ता दिनेको लिंग	
अन्तर्वार्ता दिनेको पेशा	

(परिचय)

- तपाईं र तपाईंको परिवारको बारेमा केहि भनि दिनुहोस न?

(निर्णय लिने)

- तपाईं बिरामी पर्दा कसरी तपाईंको आफ्नो ख्याल राख्नुहुन्छ ?
- के तपाई मलाई त्यस्तो समयको बारेमा बताउन सक्नुहुन्छ जब तपाईले बिरामी हुँदा स्वास्थ्य सेवा वा उपचारको बारेमा कसरी निर्णय लिनुभयो ?
- बिरामी पर्दा तपाई के बाट र कहाँ सेवा लिनु हुन्छ ?
- स्वास्थ्यकर्मी / डाक्टर / परम्परागत धामी झाँक्रीको मा उपचारको आपनो अनुभव वर्णन गरिदिनु हुन्छ कि?
- के तपाई मलाई स्वास्थ्य सेवा / निजी मेडिकल / परम्परागत धामी झाँक्रीकामा प्राप्त गर्नुभएको सेवाहरू र उपचारहरूको बारेमा बताउन सक्नुहुन्छ?

(रोग विशेष)

- तपाईलाई मलेरियाको बारेमा के थाहा छ?
- तपाईं हामीलाई आफ्नो गाउँमा मलेरियाको अनुभवको बारेमा भन्नुहोस्?
- मलेरिया तपाईं, तपाईंको गाउँमा, समुदायमा के के जोखिम ल्याउन सक्दछ ?

(जोखिम र रोकथाम)

- **के तपाईंलाई थाहा छ मलेरियाबाट पूर्ण रूपमा जोगिनको लागि कुन कुन, कस्ता खालका कदमहरू चाल्न सक्नुहुन्छ भने?**
- **सरकार**ले **मलेरिया** राकथामको लागि तपाईको **आफ्नो कुनै पनि कदम लिएको छ? तपाईको गाउँमा के भयो वर्णन गर्न सक्नुहुन्छ?**

  छलफल गर्नुहोस्: LLINs, IRS

- **य**समा **केहि समस्या / समस्याहरू** आएका **छन् ?** तपाईं तिनीहरूलाई वर्णन गर्न सक्नुहुन्छ?
