# Supplementary material for: A qualitative study of knowledge, attitudes and perceptions towards malaria prevention among people living in rural upper river valleys of Nepal
Source: PLoS One. 2022 Mar 18;17(3):e0265561. doi: 10.1371/journal.pone.0265561 (PMC8932613; doi:10.1371/journal.pone.0265561)
Supplement: Attachment — Submitted filename: Response to reviewers comments.docx [file pone.0265561.s002.docx]

**Response to reviewers**

**Manuscript title: A Qualitative Study of Knowledge, Attitude and Perceptions towards Malaria Prevention among People Living in Rural Upper River Valleys of Nepal**

**Dear reviewers, thank you for your comments and suggestions which have been addressed and colour coded in the manuscripts for your kind perusal. We would also like to acknowledge the effort and time that you have provided to go through our manuscript.**

**Reviewer 1**

**Comment 1**

This paper presented findings from only one method and source of data collection and did not mention triangulation to test the validity of the findings. While the pandemic might restrict certain data collection methods such as observation, other strategies to test validity through the convergence of information from different sources could be sought.

**Response 1**

Thank you for your suggestion. This has been now highlighted as one of the limitations of the study. Please refer to ***Page 22 Line 475-476***

As the information was collected only through virtual interviews, triangulation could not be done, a limitation of the study.

**Comment 2**

Paragraph 1 (lines 45-52):
It would be helpful for the context of the research to have epidemiological measures both nationally and in the study site for the readers to get an idea of how big of a burden is malaria in Nepal and in the study site. The first paragraph of the introduction had numbers of malaria cases, but without any denominators it is difficult to put the information in context. I would suggest adding extra information such as annual malaria incidence. Having information on the proportion of P. vivax malaria will also make the result and discussion on treatment adherence more relevant.
**Response 2**

Thank-you for the suggestion. Additional epidemiological information has been added. Please refer to ***Page 2 and 3 Line 44-48***.

Numbers of malaria cases in Nepal decreased from 11,000 in 2000 to 1,065 in 2019 and a low Annual Parasitic Incidence of 0.09 per 1,000 population at risk [3]. Among these 1065 malaria cases, 440 were indigenous (locally transmitted) and 625 were imported cases mostly from neighbouring India and countries in Africa [3]. Plasmodium vivax constituted 94.6% of the total cases in the country, whilst the remaining 5.4% were Plasmodium falciparum cases [3].

**Comment 3**

Methods
Research team (lines 105-110):
Because the researchers were not present in the study site during data collection, it is important to describe in more detail about the local research assistants, who they are, what were exactly their role in the study, and what were their relationship with the study participants.

**Response 3**

Thank-you for your suggestion. The details of the research assistants have been updated along with more information on their roles in the research. Please see page ***5 line 113***,

…local research assistants with a public health background and currently working for the national malaria program at the study site.

***Page 6 line 131-135***

Based on these criteria, a mix of potential participants were identified by the principal researcher from different cohorts that included priests, health workers, past malaria patients, teachers and students to generate rich information. The RAs supported the recruitment of the participants at the study site and arranged the necessary logistics for conducting the virtual interviews.

***Page 7 line 151-152***

Participants provided consent, both oral and written, before participating in the study and the written consent were collected by the RAs.

**Comment 4**

Participant eligibility and recruitment (lines 121-127):
I assume this research was done during the covid-19 pandemic so I expect some restrictions in contact between people. If this was the case at the study site, I think it is relevant to explain the method of approach and any non-participation.
Thank you for the comments. Additional information provided. Please refer to ***Page 6 line 131-135***

Based on these criteria, a mix of potential participants were identified by the principal researcher from different cohorts that included priests, health workers, past malaria patients, teachers and students to generate rich information. The RAs supported the recruitment of the participants at the study site and arranged the necessary logistics for conducting the virtual interviews.

**Comment 5**

Data collection (lines 137-145):
In the discussion section (line 453-454) it was mentioned that the majority of interviews were done virtually. How many were done in person and how many were done virtually?
Where were the zoom interviews held in the study site? Is it at a clinic? At participants’ own houses? Or elsewhere?

**Response 5**

Thank-you for the comment. All the interviews were conducted by the principal investigator. Additional information added, please refer to ***page 7 line 149-151*** …

While sixteen participants were interviewed in their own house, the remaining nine including health workers were interviewed in a meeting hall at the nearby health post.

... and correction made ***page 21 and 22 line 472-475***

The inclusion of participants between 15 to 72 years of age allowed us to explore the malaria prevention and treatment practices and changes across generations. Due to the COVID -19 restrictions, the interviews had to be conducted virtually.

**Comment 6**

In some of the quotes the participants mentioned their role as community health volunteers. It would be good if the results section started with a description of such characteristics. How many of the participants were community health volunteers? What about the other participants? What are their roles and positions in the society?
There is a paragraph in the discussion section about treatment adherence. Is there any data that can be added in the results section that might suggest malaria relapse or recurrence?

**Response 6**

Thank you for your comments and suggestions. Details of the participants have now been added. Please refer to ***page 8, line 174-176.***

The results of the interviews (N=25) including illustrative quotes of the participants are presented. The participants included farmers (n= 13), priest/ traditional healers (n=2), health workers (n=2), school teacher (n= 2), students (n=2), self-employed/service (n=2) and local leaders (n=2).

Regarding relapses, only general information was sought and there is no published data on malaria relapse and recurrence in Nepal. However, five participants shared their experience of relapse which has been added in the text. Please refer to ***page 10 line 202.***

However, five of the participants reported non-adherence to the treatment protocol. *“They [villagers] take medicines for 4-5 days and feel better, and then they leave the medicine as it is. We [community health volunteers] and the health workers all went there [patient’s house], convinced them, and made them take the medicines”* (female, FCHV).

**Comment 7**

Discussion
The lack of test of validity of the findings due to using only one type of data collection methods with no triangulation should be mentioned as one of the limitations.
 **Response 7**

Thank you for the suggestion. This has now been added in the manuscript under limitation, please refer to ***page 22, line 475-476***

As the information was collected through virtual interviews, triangulation could not be done, a limitation of the study.

**Comment 8**

Title and abstract
As not all readers would be familiar with places in Nepal, I personally think it might benefit the paper if the title and/or abstract mentions more descriptive words such as “remote” and/or “upper river valley.”

**Response 8**

Thank-you for your suggestion. The title of the study has been changed accordingly please refer to ***Page1 Line 1***

A Qualitative Study of Knowledge, Attitude and Perceptions towards Malaria Prevention among People Living in Rural Upper River Valleys of Nepal

**Reviewer 2**

**Comment 1**

**Methods**

Line 121 : Participant eligibility and recruitment : for in-depth interviews, participants are generally people who interact with many people in the community on a daily basis e.g. -head of districts, religious leaders, youth or women leaders etc.- as they are well informed on the population KAP… Pleased clarify if those people were included as participants. Also provide more details on the criteria used for the selection of participants.

**Response 1**

Thank you for your comments and suggestions. Details of the participants have now been added. Please refer to ***page 8, line 17r4-176***.

The results of the interviews (N=25) including illustrative quotes of the participants are presented. The participants included farmers (n= 13), priest/ traditional healers (n=2), health workers (n=2), school teacher (n= 2), students (n=2), self-employed/service (n=2) and local leaders (n=2).

**Comment 2**

Line 130 “A semi-structured interview guide was developed based on similar research [17] and the researchers’ experience exploring the topic of malaria” Can the author add as additional file the questionnaire or the guide used for the interview.
**Response 2**

Thank you very much, we have included the domains of enquiry in English for the reviewer.

**Comment 3**

Was survey done during the rainy or dry season? Please clarify as this might affect perception or risk/attitudes toward malaria prevention and treatment.

**Response 3**

Thank you for your suggestion. Some information has been added, please refer to ***Page 7 line 146-147.*** The suggestion is an interesting concept and could be a potential area for further research.

One-on-one interviews were conducted between November and December 2020 (post monsoon) by the lead author (KA) with local support from Research Assistants

**Comment 4**

Results
The author need to be more precise on the number who responded over the number interviewed.
For instance Line 183 “Most of the participants knew that they could get anti-malarial medicine from…” How many please give the number
Another Line 190 “Only half of the participants mentioned that the government had conducted…” They interviewed 25 persons what do they consider as half is it 12 or 13 people please clarify. There are many places in the document where similar terms are used they need to check the whole result section and provide full details on the number of respondent.
See again below
Line 264 : “The majority of the participants (n=10) informed that washed nets were dried in a shaded area as recommended by the manufacturer” is 10 out of 25 interviewed represents the majority ?
also see line 271 “However, most of the participants reported experiencing several negative consequences of….” Wow many please give the precise number
Line 273 “Nearly half of the participants (n=11) shared their experiences of increased bed bugs after…”. Please check and correct accordingly.

**Response 3**

Thank you for your suggestion. The information has been added ***page 9 to 14 line 186-317*** highlighted in blue.

**Comment 4**

Line 296 : I don’t see the need of adding this figure

**Response 4**

Thank you for the suggestion the figure has been removed.

**Editor’s comments**

**Comment 1**

Please ensure that your manuscript meets PLOS ONE's style requirements, including those for file naming. The PLOS ONE style templates can be found at 
<https://journals.plos.org/plosone/s/file?id=wjVg/PLOSOne_formatting_sample_main_body.pdf> and 
<https://journals.plos.org/plosone/s/file?id=ba62/PLOSOne_formatting_sample_title_authors_affiliations.pdf>

**Response**

Thank-you for the suggestion, the manuscript has been revised using the PLOS One style template

**Comment 2**

Please include additional information regarding the survey or interview guide used in the study and ensure that you have provided sufficient details that others could replicate the analyses. For instance, if you developed a survey guide as part of this study and it is not under a copyright more restrictive than CC-BY, please include a copy, in both the original language and English, as Supporting Information.

**Response 2**

Thank you very much, we have included the domains of enquiry in English.

**Comment 3**

Thank you for stating the following financial disclosure:

"The funders had no role in study design, data collection and analysis, decision to publish, or preparation of the manuscript."

At this time, please address the following queries:

a) Please clarify the sources of funding (financial or material support) for your study. List the grants or organizations that supported your study, including funding received from your institution. 
b) State what role the funders took in the study. If the funders had no role in your study, please state: “The funders had no role in study design, data collection and analysis, decision to publish, or preparation of the manuscript.”
c) If any authors received a salary from any of your funders, please state which authors and which funders.
d) If you did not receive any funding for this study, please state: “The authors received no specific funding for this work.”

Please include your amended statements within your cover letter; we will change the online submission form on your behalf.

**Response 3**

Thanks you for the information. This has been indicated in the cover letter attached with the revised submission.

**Comment 4**

We note that Figure 2 in your submission contain copyrighted images. All PLOS content is published under the Creative Commons Attribution License (CC BY 4.0), which means that the manuscript, images, and Supporting Information files will be freely available online, and any third party is permitted to access, download, copy, distribute, and use these materials in any way, even commercially, with proper attribution. For more information, see our copyright guidelines: <http://journals.plos.org/plosone/s/licenses-and-copyright>.
 
We require you to either (1) present written permission from the copyright holder to publish these figures specifically under the CC BY 4.0 license, or (2) remove the figures from your submission:
 
a. You may seek permission from the original copyright holder of Figure 2 to publish the content specifically under the CC BY 4.0 license. 
 
We recommend that you contact the original copyright holder with the Content Permission Form (<http://journals.plos.org/plosone/s/file?id=7c09/content-permission-form.pdf>) and the following text:
“I request permission for the open-access journal PLOS ONE to publish XXX under the Creative Commons Attribution License (CCAL) CC BY 4.0 (<http://creativecommons.org/licenses/by/4.0/>). Please be aware that this license allows unrestricted use and distribution, even commercially, by third parties. Please reply and provide explicit written permission to publish XXX under a CC BY license and complete the attached form.”
 
Please upload the completed Content Permission Form or other proof of granted permissions as an "Other" file with your submission. 
 
In the figure caption of the copyrighted figure, please include the following text: “Reprinted from [ref] under a CC BY license, with permission from [name of publisher], original copyright [original copyright year].”
 
b. If you are unable to obtain permission from the original copyright holder to publish these figures under the CC BY 4.0 license or if the copyright holder’s requirements are incompatible with the CC BY 4.0 license, please either i) remove the figure or ii) supply a replacement figure that complies with the CC BY 4.0 license. Please check copyright information on all replacement figures and update the figure caption with source information. If applicable, please specify in the figure caption text when a figure is similar but not identical to the original image and is therefore for illustrative purposes only.

**Response 4**

The photo was taken by the principal investigator during his visit to the study site on 25^th^ April 2018 and holds the copyright himself. However, now figure 2 has been removed.

**Comment 5**

Additional Editor Comments:

Kindly add to the limitations section within the discussion as suggested by reviewer 1, and address all comments of both reviewers.

**Response 5**

Thank you for the suggestion. The limitation section has been revised with additional information based on reviewer’s suggestion. Please refer to ***Page 21 lines 475-476.***

As the information was collected through virtual interviews, triangulation could not be done, a limitation of the study.
