# Supplementary material for: A qualitative study of knowledge, attitudes and perceptions towards malaria prevention among people living in rural upper river valleys of Nepal
Source: PLoS One. 2022 Mar 18;17(3):e0265561. doi: 10.1371/journal.pone.0265561 (PMC8932613; doi:10.1371/journal.pone.0265561)
Supplement: Attachment — Submitted filename: SCS Review of Awasthi et al. Manuscript.docx [file pone.0265561.s003.docx]

**Review of Chan et al. 2021 Manuscript**

General comments:

- Make sure your qualitative approach, methods, and analysis are all in line with one another.
- Consider using the Oxford comma throughout the manuscript.
- Would be good to have scientific editors review this for language.
- Explain the limitations in sharing raw data from manuscripts in the methods section.

Specific comments:

| Abstract | Line 29: remove “from” before females and males.  Remove “females and males” on line 30 and put “female and male” before participants. |
| --- | --- |
|  | Line 31: “the complications” explain/specify complications from or of what. |
|  | Line 32: “were provided **freely,**” freely does not mean free, would change the word to “for free” or “free of charge” |
| Introduction | Line 45: remove “to” after contributes; add “The” before numbers and remove the “s” from number. 🡪 “The number of malaria cases in Nepal decreased…” |
|  | Line 47: remove “of” before “malaria prevention interventions”; add a comma before “including”; add “the” before “free distribution” |
|  | Line 51: “all supported by greater international funding.” 🡪 what is meant by “greater” with regards to international funding? |
|  | Line 52: change “the National Malaria Strategic Plan: to “its National Malaria Strategic Plan” |
|  | Line 54: remove “to” after “contributed”; after “Geographically” add “and topographically” |
|  | Line 55: remove “further” before “divided” |
|  | Line 57: before “experience” add “, which” |
|  | Line 58: add “cross border” before “movement”; add “from Nepal” before “to India” |
|  | Line 59: after “The valleys” should “along” be substituted with “between”? Is it in between the two geographies you describe? |
|  | Line 60: change “outbreak episodes” to “outbreaks” ; Would also describe some of the epidemiological characteristics of the outbreak, including species distribution,…... |
|  | Line 63: “a village **of** Khatyad..” changed to “a village **in**…” |
|  | Line 64: change “situated” to “located”; change “URV **of**” to “URV **in**” |
|  | Line 73: remove “the” after “associated with”; |
|  | Line 74: remove of communities 🡪 Line 73+74: “Malaria is often considered a disease of the poor and has been associated with communities’ economic status and living conditions.” |
|  | Line 76: move “early” from before “health care” to after “health care” |
|  | Line 79: add a comma “,” after priorities’ change “expenditure” to “purchasing”; Line 79+80🡪 “…, and purchasing bed nets and other malaria prevention tools is not a priority.” |
|  | Line 82: change “lead to the migration of villagers to endemic urban plains in Nepal…” to “lead to villagers migrating to malaria endemic urban plains in Nepal..” + would provide more description of migration—short-term? Long-term? Daily? Weekly? Monthly? |
|  | Line 81-83: explain why this movement exposes people to an increase of malaria transmission. |
|  | Line 84: change “The” at the beginning of the sentence to “Nepal’s” |
|  | Line 86: is the “its importance” referring to regular use of the LLINs or simply the importance of LLINs? If it’s the importance of the LLINs then “its” should be changed to “their.” |
|  | Line 89: add “season” after “monsoon” |
|  | Line 92: change “repeated episodes” to “several rounds” |
|  | Line 94: Replace “leading to refusal to allow” with “leading to households refusing to allow…” ; Line 94+95 Replace “challenges to these key pillars of malaria control” to “barriers to the success of let malaria control interventions,” |
|  | Line 95: add “the” between “explore” and “knowledge”; add “of” after “knowledge”; add “about” after “attitudes” |
|  | Line 96: remove comma “,” between “treatment” and “among” |
|  | Line 97: define “wards” |
| Materials and Methods | Line 100: remove parenthesis “)” after “[21]” |
|  | Line 116: add “season” after “monsoon” ; change “**during** winter” to “**in** **the** winter” |
|  | Line 120: add a comma “,” after “’medical’” |
|  | Line 123: remove “being” before “between the ages…” |
|  | Line 125: add “their” before “roles” ; add “health related” between “in” and “decision-making” + remove “for health care” |
|  | Would be good to mention the roles/occupation of those interviewed. Also, in the results community health workers are quoted, should mention that in the paragraph on participant eligibility and recruitment. Especially, given that a phenomenological approach was taken to the qualitative research, would have to explain why health care community workers were included in the interviews if this was about high-risk population/people living in the area and their experience with malaria, its treatment, and prevention. |
|  | Line 139: remove “the” before “COVID 19 travel restrictions” |
|  | Line 140: change “zoom platform” to “Zoom video conferencing” |
|  | Line 150: add “for” after “searching” |
|  | Line 155: remove “,” comma after “organized” + add “and” after “organized” |
|  | Line 157: change “elaborate” to “develop” |
|  | Line 153: given that you are conducting a phenomenological qualitative study and you used open coding, I would add a justification for the open coding here, perhaps that you are conducting exploratory research into ideas and concepts that have not been explored in this area before. |
| Results | Figure 1: I would change the order to have the “3. Availability and Practices” third on the right, so that it reads 1,2,3 from left to right. |
|  | Line 170: change “unclear **of**” to “unclear **about**” |
|  | Line 171: “Among them” who is the “them” referring to? If it is the participants, it is not needed. |
|  | Line 178: add “malaria” before “knowledge” |
|  | Line 181: “weakness”: what is meant by weakness here? Might be good to add an illustration if their conceptualization of weakness was explored. |
|  | Line 184: describes participants having to two different types of tablets for 14 to 15 days. Might need to comment here that this is the common treatment for vivax malaria or a least for the specific type of malaria the participants had. |
|  | Line 189/190: does the female FCHV say why her patient threw away the medicine, if so, might be good to include here. |
|  | Line 204: remove semi-colin “;” |
|  | Line 211-219: description of what happened in the “past several years” 🡪 is this description something that is still happening or is it a thing of the past? If it is no longer happening, how and why did it change? |
|  | Line 220: change first two sentences to: “Traditional management of malaria was described by participants as visits to traditional healers known as ‘*Dhamis’* or ‘*Jhakris’.* However, the use of traditional healers did not seem common. The participants reported…” |
|  | Line 226: look like a main header, should be under the ‘choices and preferences’ header right? |
|  | Line 239: remove “and journey of getting” and replace with “of the journey to get” |
|  | Line 242: move the USD cost before the ending quotation mark. |
|  | Line 244: change “the treatment options” to “where treatment is sought” |
|  | Line 245: change “for” to “regarding” + add “matters” after “health” |
|  | Line 252: division of what? \| remove “the” before “LLINS” |
|  | Line 255: remove “round” after “year” |
|  | Line 258: add a comma “,” after detergents and add “baking” after soda otherwise need to explain what is meant by soda here. |
|  | Line 261: add a comma “,” between “nets” and “and” |
|  | Line 265: change “informed” to “mentioned” |
|  | Line 272: change “in the households” to “in their houses.” |
|  | Line 283: add “also” after “participants” ; change “the family business of bee-farming” to “bee-farming family businesses” |
|  | Line 288: remove comma “,” after bees |
|  | Line 293: “several participants in the community” 🡪 are these interview participants? |
| Discussion | Line 300: change “knowledge on” to “knowledge of” ; “However,” might not be necessary to start the second sentence on that line. |
|  | Line 303: “in the area” 🡪 what is meant by in the area? Which area? |
|  | Line 304: add “malaria” before “prevention and treatment” |
|  | Line 312: what is consistent with research by Yadaw et al? “90% of respondents” 🡪 whose respondents are these—which study is this from. Not clear. |
|  | Line 314: change “the higher SES” to “those of higher SES” |
|  | Line 316: how are you defining lower SES group? What want to consider saying “are of low socio-economic status, but make sure to define. |
|  | Line 320: add “treatment” before “protocol” |
|  | Line 329: is this how the patients described their side effects? Anorexia and allergic reactions. Seems like pretty technical language. |
|  | Line 330: the trial talked about recurrence, not relapse. Would also add in the dosage information from the trial. |
|  | Line 350/351: “Malaria treatment is free in public health facilities across Nepal, a reason that could explain that preference.” Isn’t it the case that participants provided the reasoning for their preference of public health facilities? Would be careful how you formulate this sentence. |
|  | Line 352/353: description of traditional healing as still being an option. Is not quite in alignment with how you report it in the result section. |
|  | Line 354: change “acceptance **to**” to “acceptance **of**” |
|  | Line 355: change “(western)” “(western-style)” |
|  | Line 363: change “limitations **in**” to “limitations **of**” |
|  | Line 368: remove “completeness” |
|  | Line 369: change to “, due to weak monitoring and lack of commercially available Primaquine in Nepal [31].” |
|  | Line 373: countries like Bangladesh or actually Bangladesh? + remove “in” after “among” |
|  | Line 374: change “have no reporting access to the national…” to “cannot report to the national….” |
|  | Line 375: change “resulting in missed cases” to “resulting in the surveillance system missing cases.” |
|  | Line 377-380: feels like this sentence comes out nowhere. Would provide the findings this recommendation is based on first then provide the recommendation. |
|  | Line 382: add “at” between “treatment” and “facilities” |
|  | Line 383: change “this” to “Such accessibility and availability” |
|  | Line 384: explain why it would be crucial to prevent local transmission or outbreaks. |
|  | Line 389: Add comma “,” after “insurance system” and after “(OPP)” |
|  | Line 390: add “seeking” after “health care” |
|  | Line 892: remove “the” before “physical” + remove “s” at the end of “conditions” + change “and” to “or” before “old age” |
|  | Line 394: change “transport **facilities**” to “transport **infrastructure**” |
|  | Line 398: change “saving them” to “mitigating” |
|  | Line 400: change “for a” after “known” to “as a” |
|  | Line 401: add a comma “,” after “society” + change “taken” to “made” after “were” |
|  | Lines 407-409: where is the description? |
|  | Line 418: Remove “The” before “LLINs” change “a factor **for**” to “a factor **in**” |
|  | Line 420: add “a” before “high risk of malaria” + remove comma “,” after “malaria” |
|  | Line 421: remove “the” before “private” |
|  | Line 422: change “access **to**” to “access **for**” |
|  | Line 423: add “Additionally,” before “Normal nets” |
|  | Line 426: add “baking” before “soda” + change “for washing the LLINs” to “to wash LIINs” + remove the comma “,” after “LLINs”. |
|  | Line 428: change “for washing” to “to wash” |
|  | Line 431: change “negative consequences of IRS” to “unintended negative consequences from IRS” |
|  | Line 433: change “barriers **for**” to “barriers **to**” |
|  | Line 434: change “shifting” to “moving” before “the beehives” + add an “s” to “outdoor” + change “in” to “to” after “outdoor” |
|  | Line 439: add “from our findings.” after “differed” + end the sentence there and start a new one with “Barriers identified by respondents in xxxx included people believing it increased rodents….” 🡪 need to also add context to these barriers i.e., location. |
|  | Line 443: examples of political factors. |
|  | Line 443: first word, change “for building” to “creating” |
|  | Line 445: add “described by our participants” after “negative experiences” |
|  | Line 446: change “albeit” to “while” |
| Limitations | Line 450: “the local community” 🡪 specify which local community. |
|  | Line 452: remove “the” before “malaria prevention” |
|  | Line 453: remove “the” before “COVID-19 restrictions” |
|  | Line 455: add “malaria” before “endemic” |
|  | Line 456: change “people **on**” to “people **relating to**” |
| Conclusion | Line 463: “people in rural areas to seek alternative care from traditional healers and private medicals.” 🡪 This isn’t in agreement with your results. Would make this more nuanced so it is in line with the reporting of results. |
