# Supplementary material for: A qualitative study of knowledge, attitudes and perceptions towards malaria prevention among people living in rural upper river valleys of Nepal
Source: PLoS One. 2022 Mar 18;17(3):e0265561. doi: 10.1371/journal.pone.0265561 (PMC8932613; doi:10.1371/journal.pone.0265561)
Supplement: Attachment — Submitted filename: Response to reviewers.docx [file pone.0265561.s004.docx]

**/Reviewers Response**

**Manuscript title: Title:** A Qualitative Study of Knowledge, Attitudes and Perceptions towards Malaria Prevention among People Living in Khatyad Rural Municipality of Mugu, Nepal

Dear reviewers, thank you for your comments and suggestions which have been addressed and track changed in the manuscript for your kind perusal. We would also like to acknowledge the effort and time that you have provided to go through our manuscript.

Reviewer 1

**Comment 1**

There is a paragraph about treatment adherence (lines 335-352) in the discussion section. However, I do not see this being a prominent finding in the results section. I would suggest to put more data about treatment adherence issues in the result section to justify this paragraph or remove/reduce discussion about treatment adherence issue (perhaps as part of malaria treatment knowledge?).

**Response 1**

Thank you for your comments and suggestion. There is a paragraph on treatment adherence in the results section please refer to page 11 line 216-222. Some additional information has also been added. Knowledge and practice of drug adherence was an important finding in the study, particularly due to the fact that *Plasmodium vivax* is the predominant species in the area.

**Comment 2**

Socioeconomic status (SES) was mentioned and discussed in the paragraph regarding malaria knowledge (lines 322-334). In my view, this is also relevant in the later paragraph discussing access to treatment (380-401). While this manuscript is not focused on intersectionality between different factors, I think it will benefit the manuscript to bring readers' attention to the complexity of malaria-related problem in this setting.

**Response 2**

Thank you for your comments. As you have correctly indicated, the paper is not focused on intersectionality. The study explored the underlying factors of how SES may be associated with poor knowledge and reluctance to seek appropriate health care (please refer to page 16 line 345- 346).

**Comment 3**

Virtual interview is mentioned as a limitation of this study. In the current world we are living in, virtual interview and even virtual ethnography might be explored more. Is there anything readers could learn from your experience doing the virtual interviews? In your opinion, is this method of data collection have any influence on the results? Would the results of the interview be any different (apart from lack of 'rich' data and ability to triangulate) had it been done in person? How has the study participants react to their involvement in a study that uses this kind of technology? What was the measures taken to ensure confidentiality and anonymity, since I assume the recorded version of the interviews include video showing the faces of the study participants? The answers to these questions might worth a paragraph in the discussion section.

**Response 3**

Thank you very much for this is an interesting observation. The virtual interviews compromised the discussion slightly due to poor network, however we still felt the interaction was successful and very similar to conducting a face to face interview. Please refer to page 23 line 508-511. Only audio recordings were saved (page 8 line 170) and anonymity was maintained by using pseudonyms and removing any personal identifiers (page 8 line 171-173).

**Comment 4**
In relation to point #3, I think a paragraph needs to be added in the methods section explaining confidentiality and anonymity, including who have access to the recorded file and where it is stored.

**Response 4**

Thank you very much for your comments. This research has been approved by two ethical research committees, (i.e. NHRC ethics of Nepal and HREC of Curtin University) and anonymity and confidentiality have been strictly adhered to. All the raw data has been securely stored in the Curtin university HREC repository and this has been added in the manuscript. Please refer to page 8 line 172.

**Comment 5**

In the abstract, I would prefer to use "in-depth interviews" rather than "one-on-one interviews" (line 26) as that is the common term and is what is stated in the methods section (line 138). Or perhaps "virtual in-depth interviews"?

**Response 5**

Thank you for the suggestion. The one-on-one interviews have been changed to in-depth interviews as suggested (page 2 line 29 and page 8 line 162)

Reviewer 3

| **Section** | **Reviewers Comments** | **Response** |
| --- | --- | --- |
| **Thank you for your meticulous effort in providing editorial feedback on the manuscript which is highly appreciated. We have corrected the grammatical errors and punctuations as per the suggestion.** | | |
| **Abstract** | Line 29: remove “from” before females and males.  Remove “females and males” on line 30 and put “female and male” before participants. | Thank you for your suggestion. Correction has been made as suggested. Please refer to page 2 line 30 |
|  | Line 31: “the complications” explain/specify complications from or of what. | Thank you for your suggestion. Correction has been made as suggested. Please refer to page 2 line 32 |
|  | Line 32: “were provided **freely,**” freely does not mean free, would change the word to “for free” or “free of charge” | Thank you for your suggestion. Correction has been made as suggested. Please refer to page 2 line 33 |
| **Introduction** | Line 45: remove “to” after contributes; add “The” before numbers and remove the “s” from number. 🡪 “The number of malaria cases in Nepal decreased…” | Thank you for your suggestion. Correction has been made as suggested. Please refer to page 3 line 46 |
|  | Line 47: remove “of” before “malaria prevention interventions”; add a comma before “including”; add “the” before “free distribution” | Thank you for your suggestion. Correction has been made as suggested. Please refer to page 3 line 53 |
|  | Line 51: “all supported by greater international funding.” 🡪 what is meant by “greater” with regards to international funding? | Thank you for your suggestion. Correction has been made as suggested. Please refer to page 3 line 56 |
|  | Line 52: change “the National Malaria Strategic Plan: to “its National Malaria Strategic Plan” | Thank you for your suggestion. Correction has been made as suggested. Please refer to page 3 line 58 |
|  | Line 54: remove “to” after “contributed”; after “Geographically” add “and topographically” | Thank you for your suggestion. Correction has been made as suggested. Please refer to page 3 line 61,62 |
|  | Line 55: remove “further” before “divided” | Thank you for your suggestion. Correction has been made as suggested. Please refer to page 3 line 62 |
|  | Line 57: before “experience” add “, which” | Thank you for your suggestion. Correction has been made as suggested. Please refer to page 3 line 64 |
|  | Line 58: add “cross border” before “movement”; add “from Nepal” before “to India” | Thank you for your suggestion. Correction has been made as suggested. Please refer to page 3 line 65 |
|  | Line 59: after “The valleys” should “along” be substituted with “between”? Is it in between the two geographies you describe? | Thank you for your suggestion. Correction has been made as suggested. Please refer to page 3 line 66 |
|  | Line 60: change “outbreak episodes” to “outbreaks” ; Would also describe some of the epidemiological characteristics of the outbreak, including species distribution,…... | Thank you for your suggestion. Correction has been made as suggested. Additional information added to bring clarity to cases. Please refer to page 3 line 68 and page 4 line 70 |
|  | Line 63: “a village **of** Khatyad.” changed to “a village **in**…” | Thank you for your suggestion. Correction has been made as suggested. Please refer to page 4 line 71 |
|  | Line 64: change “situated” to “located”; change “URV **of**” to “URV **in**” | Thank you for your suggestion. Correction has been made as suggested. Please refer to page 4 line 72 |
|  | Line 73: remove “the” after “associated with”; | Thank you for your suggestion. Correction has been made as suggested. Please refer to page 4 line 81 |
|  | Line 74: remove of communities 🡪 Line 73+74: “Malaria is often considered a disease of the poor and has been associated with communities’ economic status and living conditions.” | Thank you for your suggestion. Correction has been made as suggested. Please refer to page 4 line 82 |
|  | Line 76: move “early” from before “health care” to after “health care” | Thank you for your suggestion. Correction has been made as suggested. Please refer to page 4 line 84-85 |
|  | Line 79: add a comma “,” after priorities’ change “expenditure” to “purchasing”; Line 79+80🡪 “…, and purchasing bed nets and other malaria prevention tools is not a priority.” | Thank you for your suggestion. Correction has been made as suggested. Please refer to page 4 line 82, 87 |
|  | Line 82: change “lead to the migration of villagers to endemic urban plains in Nepal…” to “lead to villagers migrating to malaria endemic urban plains in Nepal..” + would provide more description of migration—short-term? Long-term? Daily? Weekly? Monthly? | Thank you for the suggestion. The editing has been done as per advice and addition clarity in information has been added for the case characteristics. Please refer to page 4 and 5 line 89, 91-93 |
|  | Line 81-83: explain why this movement exposes people to an increase of malaria transmission. | Thank you for your suggestion. Correction has been made as suggested. Please refer to page 4 and 5 line 91-93 |
|  | Line 84: change “The” at the beginning of the sentence to “Nepal’s” | Thank you for your suggestion. Correction has been made as suggested. Please refer to page 5 line 95 |
|  | Line 86: is the “its importance” referring to regular use of the LLINs or simply the importance of LLINs? If it’s the importance of the LLINs then “its” should be changed to “their.” | Thank you for your suggestion. Correction has been made as suggested. Please refer to page 5 line 97 |
|  | Line 89: add “season” after “monsoon” | Thank you for your suggestion. Correction has been made as suggested. Please refer to page 5 line 100 |
|  | Line 92: change “repeated episodes” to “several rounds” | Thank you for your suggestion. Correction has been made as suggested. Please refer to page 5 line 104 |
|  | Line 94: Replace “leading to refusal to allow” with “leading to households refusing to allow…” ; Line 94+95 Replace “challenges to these key pillars of malaria control” to “barriers to the success of let malaria control interventions,” | Thank you for your suggestion. Correction has been made as suggested. Please refer to page 5 line 105-107 |
|  | Line 95: add “the” between “explore” and “knowledge”; add “of” after “knowledge”; add “about” after “attitudes” | Thank you for your suggestion. Correction has been made as suggested. Please refer to page 5 line 108 |
|  | Line 96: remove comma “,” between “treatment” and “among” | Thank you for your suggestion. Correction has been made as suggested. Please refer to page 5 line 109 |
|  | Line 97: define “wards” | Thank you for your suggestion. Correction has been made as suggested. Please refer to page 5 line 110 |
| **Materials and Methods** | Line 100: remove parenthesis “)” after “[21]” | Thank you for your suggestion. Correction has been made as suggested. Please refer to page 6 line 113 |
|  | Line 116: add “season” after “monsoon” ; change “**during** winter” to “**in** **the** winter” | Thank you for your suggestion. Correction has been made as suggested. Please refer to page 6 line 132 |
|  | Line 120: add a comma “,” after “’medical’” | Thank you for your suggestion. Correction has been made as suggested. Please refer to page 6 line 135 |
|  | Line 123: remove “being” before “between the ages…” | Thank you for your suggestion. Correction has been made as suggested. Please refer to page 7 line 139 |
|  | Line 125: add “their” before “roles” ; add “health related” between “in” and “decision-making” + remove “for health care” | Thank you for your suggestion. Correction has been made as suggested. Please refer to page 7 line 141 |
|  | Would be good to mention the roles/occupation of those interviewed. Also, in the results community health workers are quoted, should mention that in the paragraph on participant eligibility and recruitment. Especially, given that a phenomenological approach was taken to the qualitative research, would have to explain why health care community workers were included in the interviews if this was about high-risk population/people living in the area and their experience with malaria, its treatment, and prevention. | Thank you for your suggestion. Additional information has been added as suggested. Please refer to page 7 line 142-146 |
|  | Line 139: remove “the” before “COVID 19 travel restrictions” | Thank you for your suggestion. Correction has been made as suggested. Please refer to page 8 line 164 |
|  | Line 140: change “zoom platform” to “Zoom video conferencing” | Thank you for your suggestion. Correction has been made as suggested. Please refer to page 8 line 165 |
|  | Line 150: add “for” after “searching” | Thank you for your suggestion. Correction has been made as suggested. Please refer to page 8 line 178 |
|  | Line 155: remove “,” comma after “organized” + add “and” after “organized” | Thank you for your suggestion. Correction has been made as suggested. Please refer to page 8 line 184 |
|  | Line 157: change “elaborate” to “develop” | Thank you for your suggestion. Correction has been made as suggested. Please refer to page 9 line 186 |
|  | Line 153: given that you are conducting a phenomenological qualitative study and you used open coding, I would add a justification for the open coding here, perhaps that you are conducting exploratory research into ideas and concepts that have not been explored in this area before. | Thank you for your suggestion. Additional information has been added as per the suggestion. Please refer to page 8 line 181 |
| **Results** | Figure 1: I would change the order to have the “3. Availability and Practices” third on the right, so that it reads 1,2,3 from left to right. | Thank you for your suggestion. Correction has been made to the figures suggested. Please refer to page 10 line 198-199 |
|  | Line 170: change “unclear **of**” to “unclear **about**” | Thank you for your suggestion. Correction has been made as suggested. Please refer to page 10 line 203 |
|  | Line 171: “Among them” who is the “them” referring to? If it is the participants, it is not needed. | Thank you for your suggestion. Correction has been made as suggested. Please refer to page 10 line 204 |
|  | Line 178: add “malaria” before “knowledge” | Thank you for your suggestion. Correction has been made as suggested. Please refer to page 11 line 211 |
|  | Line 181: “weakness”: what is meant by weakness here? Might be good to add an illustration if their conceptualization of weakness was explored. | Thank you for your suggestion. Correction has been made as suggested. Please refer to page 11 line 214 |
|  | Line 184: describes participants having to two different types of tablets for 14 to 15 days. Might need to comment here that this is the common treatment for vivax malaria or a least for the specific type of malaria the participants had. | Thank you for your suggestion. Correction has been made as suggested. Please refer to page 11 line 218 |
|  | Line 189/190: does the female FCHV say why her patient threw away the medicine, if so, might be good to include here. | Thank you for your suggestion. Some additional information has been added as per the suggestion. Please refer to page 11 line 221,222 |
|  | Line 204: remove semi-colin “;” | Thank you for your suggestion. Correction has been made as suggested. Please refer to page 12 line 240 |
|  | Line 211-219: description of what happened in the “past several years” 🡪 is this description something that is still happening or is it a thing of the past? If it is no longer happening, how and why did it change? | Thank you for your suggestion. Correction has been made as suggested. Please refer to page 12 line 247 |
|  | Line 220: change first two sentences to: “Traditional management of malaria was described by participants as visits to traditional healers known as ‘*Dhamis’* or ‘*Jhakris’.* However, the use of traditional healers did not seem common. The participants reported…” | Thank you for your suggestion. Correction has been made as suggested. Please refer to page 13 line 256-258 |
|  | Line 226: look like a main header, should be under the ‘choices and preferences’ header right? | Thank you for your suggestion. Correction has been made as suggested. Please refer to page 13 line 264 |
|  | Line 239: remove “and journey of getting” and replace with “of the journey to get” | Thank you for your suggestion. Correction has been made as suggested. Please refer to page 14 line 277 |
|  | Line 242: move the USD cost before the ending quotation mark. | Thank you for your suggestion. Correction has been made as suggested. Please refer to page 14 line 280 |
|  | Line 244: change “the treatment options” to “where treatment is sought” | Thank you for your suggestion. Correction has been made as suggested. Please refer to page 14 line 282 |
|  | Line 245: change “for” to “regarding” + add “matters” after “health” | Thank you for your suggestion. Correction has been made as suggested. Please refer to page 14 line 284 |
|  | Line 252: division of what? \| remove “the” before “LLINS” | Thank you for your suggestion. Correction has been made as suggested. Please refer to page 14 line 291. EDCD is a proper noun and is a section under the Ministry of Health in Nepal that oversees communicable and non-communicable diseases. |
|  | Line 255: remove “round” after “year” | Thank you for your suggestion. Correction has been made as suggested. Please refer to page 14 line 294 |
|  | Line 258: add a comma “,” after detergents and add “baking” after soda otherwise need to explain what is meant by soda here. | Thank you for your suggestion. Correction has been made as suggested. Please refer to page 14 line 298 |
|  | Line 261: add a comma “,” between “nets” and “and” | Thank you for your suggestion. Correction has been made as suggested. Please refer to page 15 line 302 |
|  | Line 265: change “informed” to “mentioned” | Thank you for your suggestion. Correction has been made as suggested. Please refer to page 15 line 305 |
|  | Line 272: change “in the households” to “in their houses.” | Thank you for your suggestion. Correction has been made as suggested. Please refer to page 15 line 312 |
|  | Line 283: add “also” after “participants” ; change “the family business of bee-farming” to “bee-farming family businesses” | Thank you for your suggestion. Correction has been made as suggested. Please refer to page 16 line 323 |
|  | Line 288: remove comma “,” after bees | Thank you for your suggestion. Correction has been made as suggested. Please refer to page 16 line 328 |
|  | Line 293: “several participants in the community” 🡪 are these interview participants? | Thank you for your suggestion. These are the interview participants. Correction has been made as suggested. Please refer to page 16 line 333 |
| Discussion | Line 300: change “knowledge on” to “knowledge of” ; “However,” might not be necessary to start the second sentence on that line. | Thank you for your suggestion. Correction has been made as suggested. Please refer to page 16 line 339 |
|  | Line 303: “in the area” 🡪 what is meant by in the area? Which area? | Thank you for your suggestion. Correction has been made as suggested. Please refer to page 16 line 342 |
|  | Line 304: add “malaria” before “prevention and treatment” | Thank you for your suggestion. Correction has been made as suggested. Please refer to page 16 line 343 |
|  | Line 312: what is consistent with research by Yadav et al? “90% of respondents” 🡪 whose respondents are these—which study is this from. Not clear. | Thank you for your suggestion. Correction has been made as suggested. Please refer to page 17 line 353 |
|  | Line 314: change “the higher SES” to “those of higher SES” | Thank you for your suggestion. Correction has been made as suggested. Please refer to page 17 line 355 |
|  | Line 316: how are you defining lower SES group? What want to consider saying “are of low socio-economic status, but make sure to define. | Thank you for your suggestion. The lower SES has been rephrased as per the suggestion. Please refer to page line 6 line 129 under methods. The referred study Yadav et al. conducted in Rajasthan of India does not clearly state the SES categories. |
|  | Line 320: add “treatment” before “protocol” | Thank you for your suggestion. Correction has been made as suggested. Please refer to page 17 line 363 |
|  | Line 329: is this how the patients described their side effects? Anorexia and allergic reactions. Seems like pretty technical language. | Thank you for your suggestion. Correction has been made as suggested. Please refer to page 18 line 371-372 |
|  | Line 330: the trial talked about recurrence, not relapse. Would also add in the dosage information from the trial. | Thank you for your suggestion. Correction has been made as suggested and additional information on dosage has been added. Please refer to page 18 line 373-375 |
|  | Line 350/351: “Malaria treatment is free in public health facilities across Nepal, a reason that could explain that preference.” Isn’t it the case that participants provided the reasoning for their preference of public health facilities? Would be careful how you formulate this sentence. | Thank you for your suggestion. Correction has been made as suggested. Please refer to page 19 line 395 |
|  | Line 352/353: description of traditional healing as still being an option. Is not quite in alignment with how you report it in the result section. | Thank you for your suggestion. Correction has been made as suggested. Please refer to page 19 line 396-398 |
|  | Line 354: change “acceptance **to**” to “acceptance **of**” | Thank you for your suggestion. Correction has been made as suggested. Please refer to page 19 line 399 |
|  | Line 355: change “(western)” “(western-style)” | Thank you for your suggestion. Correction has been made as suggested. Please refer to page 19 line 399 |
|  | Line 363: change “limitations **in**” to “limitations **of**” | Thank you for your suggestion. Correction has been made as suggested. Please refer to page 19 line 408 |
|  | Line 368: remove “completeness” | Thank you for your suggestion. Correction has been made as suggested. Please refer to page 19 line 413 |
|  | Line 369: change to “, due to weak monitoring and lack of commercially available Primaquine in Nepal [31].” | Thank you for your suggestion. Correction has been made as suggested. Please refer to page 19 line 414 |
|  | Line 373: countries like Bangladesh or actually Bangladesh? + remove “in” after “among” | Thank you for your suggestion. Correction has been made as suggested. Please refer to page 20 line 418 |
|  | Line 374: change “have no reporting access to the national…” to “cannot report to the national….” | Thank you for your suggestion. Correction has been made as suggested. Please refer to page 20 line 419 |
|  | Line 375: change “resulting in missed cases” to “resulting in the surveillance system missing cases.” | Thank you for your suggestion. Correction has been made as suggested. Please refer to page 20 line 420-421 |
|  | Line 377-380: feels like this sentence comes out nowhere. Would provide the findings this recommendation is based on first then provide the recommendation. | Thank you for your suggestion. This has been removed. Please refer to page 20 line 429-433 |
|  | Line 382: add “at” between “treatment” and “facilities” | Thank you for your suggestion. Correction has been made as suggested. Please refer to page 20 line 428 |
|  | Line 383: change “this” to “Such accessibility and availability” | Thank you for your suggestion. Correction has been made as suggested. Please refer to page 20 line 429 |
|  | Line 384: explain why it would be crucial to prevent local transmission or outbreaks. | Thank you for your suggestion. Correction has been made as suggested. Please refer to page 20 line 430-433 |
|  | Line 389: Add comma “,” after “insurance system” and after “(OPP)” | Thank you for your suggestion. Correction has been made as suggested. Please refer to page 20 line 438 |
|  | Line 390: add “seeking” after “health care” | Thank you for your suggestion. Correction has been made as suggested. Please refer to page 20 line 439 |
|  | Line 892: remove “the” before “physical” + remove “s” at the end of “conditions” + change “and” to “or” before “old age” | Thank you for your suggestion. Correction has been made as suggested. Please refer to page 21 line 441 |
|  | Line 394: change “transport **facilities**” to “transport **infrastructure**” | Thank you for your suggestion. Correction has been made as suggested. Please refer to page 21 line 443 |
|  | Line 398: change “saving them” to “mitigating” | Thank you for your suggestion. Correction has been made as suggested. Please refer to page 21 line 447 |
|  | Line 400: change “for a” after “known” to “as a” | Thank you for your suggestion. Correction has been made as suggested. Please refer to page 21 line 434 |
|  | Line 401: add a comma “,” after “society” + change “taken” to “made” after “were” | Thank you for your suggestion. Correction has been made as suggested. Please refer to page 21 line 450-451 |
|  | Lines 407-409: where is the description? | Thank you for your suggestion. The description is provided in the paragraph starting page 21 line 452 and 459 |
|  | Line 418: Remove “The” before “LLINs” change “a factor **for**” to “a factor **in**” | Thank you for your suggestion. Correction has been made as suggested. Please refer to page 22 line 469 |
|  | Line 420: add “a” before “high risk of malaria” + remove comma “,” after “malaria” | Thank you for your suggestion. Correction has been made as suggested. Please refer to page 22 line 471 |
|  | Line 421: remove “the” before “private” | Thank you for your suggestion. Correction has been made as suggested. Please refer to page 22 line 472 |
|  | Line 422: change “access **to**” to “access **for**” | Thank you for your suggestion. Correction has been made as suggested. Please refer to page 22 line 473 |
|  | Line 423: add “Additionally,” before “Normal nets” | Thank you for your suggestion. Correction has been made as suggested. Please refer to page 22 line 474 |
|  | Line 426: add “baking” before “soda” + change “for washing the LLINs” to “to wash LIINs” + remove the comma “,” after “LLINs”. | Thank you for your suggestion. Correction has been made as suggested. Please refer to page 22 line 477 |
|  | Line 428: change “for washing” to “to wash” | Thank you for your suggestion. Correction has been made as suggested. Please refer to page 22 line 479 |
|  | Line 431: change “negative consequences of IRS” to “unintended negative consequences from IRS” | Thank you for your suggestion. Correction has been made as suggested. Please refer to page 22 line 483 |
|  | Line 433: change “barriers **for**” to “barriers **to**” | Thank you for your suggestion. Correction has been made as suggested. Please refer to page 22 line 485 |
|  | Line 434: change “shifting” to “moving” before “the beehives” + add an “s” to “outdoor” + change “in” to “to” after “outdoor” | Thank you for your suggestion. Correction has been made as suggested. Please refer to page 22 line 486, 487 |
|  | Line 439: add “from our findings.” after “differed” + end the sentence there and start a new one with “Barriers identified by respondents in xxxx included people believing it increased rodents….” 🡪 need to also add context to these barriers i.e., location. | Thank you for your suggestion. Correction has been made as suggested. Please refer to page 23 line 492,493 |
|  | Line 443: examples of political factors. | Thank you for your suggestion. Correction has been made as suggested. Please refer to page 23 line 497 |
|  | Line 443: first word, change “for building” to “creating” | Thank you for your suggestion. Correction has been made as suggested. Please refer to page 23 line 498 |
|  | Line 445: add “described by our participants” after “negative experiences” | Thank you for your suggestion. Correction has been made as suggested. Please refer to page 23 line 500 |
|  | Line 446: change “albeit” to “while” | Thank you for your suggestion. Correction has been made as suggested. Please refer to page 23 line 501 |
| Limitations | Line 450: “the local community” 🡪 specify which local community. | Thank you for your suggestion. Correction has been made as suggested. Please refer to page 23 line 504 |
|  | Line 452: remove “the” before “malaria prevention” | Thank you for your suggestion. Correction has been made as suggested. Please refer to page 23 line 507 |
|  | Line 453: remove “the” before “COVID-19 restrictions” | Thank you for your suggestion. Correction has been made as suggested. Please refer to page 23 line 508 |
|  | Line 455: add “malaria” before “endemic” | Thank you for your suggestion. Correction has been made as suggested. Please refer to page 24 line 514 |
|  | Line 456: change “people **on**” to “people **relating to**” | Thank you for your suggestion. Correction has been made as suggested. Please refer to page 24 line 515 |
| Conclusion | People in rural areas to seek alternative care from traditional healers and private medicals.” -> This isn’t in agreement with your results. Would make this more nuanced so it is in line with the reporting of results. | Thank you for your suggestion. Correction has been made as suggested. Please refer to page 23 line 521 |
